# A system biology approach highlights a hormonal enhancer effect on regulation of genes in a nitrate responsive "biomodule"

**DOI:** 10.1186/1752-0509-3-59

**Published:** 2009-06-06

**Authors:** Damion Nero, Gabriel Krouk, Daniel Tranchina, Gloria M Coruzzi

**Affiliations:** 1 Center for Genomics and Systems Biology, Department of Biology, New York University, 100 Washington Square East, 1009 Main Building, New York, 10003, USA; 2 Courant Institute of Mathematical Sciences, New York, 251 Mercer St, New York, NY, 10012, USA

## Abstract

**Background:**

Nitrate-induced reprogramming of the transcriptome has recently been shown to be highly context dependent. Herein, a systems biology approach was developed to identify the components and role of cross-talk between nitrate and hormone signals, likely to be involved in the conditional response of NO_3_^- ^signaling.

**Results:**

Biclustering was used to identify a set of genes that are N-responsive across a range of Nitrogen (N)-treatment backgrounds (i.e. nitrogen treatments under different growth conditions) using a meta-dataset of 76 Affymetrix ATH1 chips from 5 different laboratories. Twenty-one biclusters were found to be N-responsive across subsets of this meta-dataset. *N-bicluster 9 *(126 genes) was selected for further analysis, as it was shown to be reproducibly responsive to NO_3_^- ^as a signal, across a wide-variety of background conditions and datasets. *N-bicluster 9 *genes were then used as "seed" to identify putative cross-talk mechanisms between nitrate and hormone signaling. For this, the 126 nitrate-regulated genes in *N-bicluster 9 *were biclustered over a meta-dataset of 278 ATH1 chips spanning a variety of hormone treatments. This analysis divided the bicluster 9 genes into two classes: i) genes controlled by NO_3_^- ^only *vs*. ii) genes controlled by *both *NO_3_^- ^and hormones. The genes in the latter group showed a NO_3_^- ^response that is significantly enhanced, compared to the former. *In silico *analysis identified two Cis-Regulatory Elements candidates (CRE) (E2F, HSE) potentially involved the interplay between NO_3_^- ^and hormonal signals.

**Conclusion:**

This systems analysis enabled us to derive a hypothesis in which hormone signals are proposed to enhance the nitrate response, providing a potential mechanistic explanation for the link between nitrate signaling and the control of plant development.

## Background

Higher plants acquire nitrogen mainly as NO_3_^-^. The soil concentration of this mineral ion can fluctuate dramatically in the rhizosphere, often resulting in limited growth and yield [1]. Thus, nitrate signaling constitutes a key point of plant adaptation to environment. This is why nitrate signaling has so far been intensively studied by transcriptomic assays, involving more than 75 ATH1 chips in various background conditions and treatments. Taken together these transcriptomic data showed that NO_3_^-^-responses are very context dependent [2,3], suggesting that evolution probably built very adaptable and robust networks involved in the integration of NO_3_^- ^with other signals including light, sugar, and hormones. For instance, as sessile organisms, plants have developed a strong capacity to modulate growth according to nutrient availability. On a molecular scale, this coordination between nutrition and growth can be mediated by the co-control of metabolism and hormonal signaling. For instance, a recent work reports that molecular reprogramming induced by nutritional starvation treatments significantly involve hormone regulated genes [4]. Moreover, it has also been shown that such cross-controls exist between NO_3_^- ^and: cytokinin (for review see [5]), auxin [2,6,7], and ABA [8]. To date, molecular players underlying those events are still under investigation. One striking example of such coordination at a molecular level is presented by the role of the iso-pentenyl-transferase 3 (IPT3) involved in the critical step of cytokinin biosynthesis. Transcription of the NO_3_^- ^induced gene IPT3 has been shown to be involved in the production of NO_3_^- ^induced cytokinins, hypothesized to coordinate shoot growth in response to NO_3_^- ^provision [9-14].

Root architecture is also under the coordinated control of nutrient availability and hormone signaling [15]. For instance, NO_3_^- ^controls root branching under various pathways (for review see [16,17]). Hormones have been shown to play important roles in the adaptation of root development to NO_3_^- ^availability. Indeed, NO_3_^- ^triggers root colonization in NO_3_^- ^rich patch of the soil. Zhang et al [18] have shown that this adaptation could involve AXR4, a gene initially demonstrated to be involved in auxin signaling. Later, AXR4 was shown to be involved in targeting the auxin influx transporter AUX1 to the plasma membrane [19]. Thus AXR4 may provide a molecular link between the NO_3_^- ^signal and auxin signaling through regulating auxin transport. Furthermore, the dual affinity (high and low affinity NO_3_^- ^uptake) NO_3_^- ^transporter NRT1.1/CHL1, hypothesized to be a part of the NO_3_^- ^sensing system [20-23], was previously shown to be regulated by auxin [7]. This evidence uncovers one facet of how the NO_3_^- ^sensing system is likely tuned by a hormonal/growth signal.

The complexity of the NO_3_^- ^effect on root development is further complicated by the fact that high NO_3_^- ^concentrations (50 mM) trigger an almost complete repression of the lateral root development (LRD). Abscisic acid (ABA) seems to be required for this effect, since the NO_3_^- ^inhibitory effect on LRD is reduced by mutating either the ABI4 or ABI5 genes [8].

**Figure 1:**
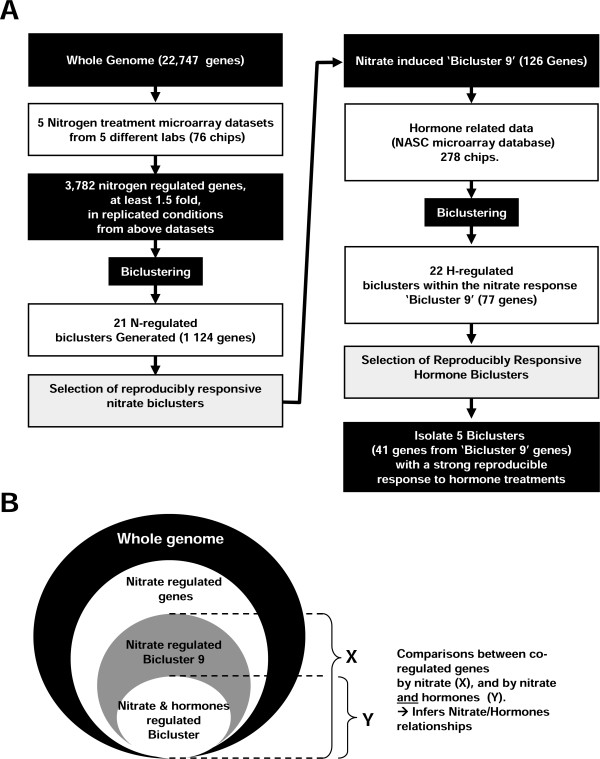
**Schematic representation of the *in silico *strategy used to decipher a N/hormone crosstalk module**. (A) The strategy was to discover via sequential biclustering filtering stages, co-regulated genes in response to: i) N treatments, and also ii) hormone treatments. (B) This delimitation of gene groups (whole genome, NO_3_^-^-regulated, Bicluster9 NO_3_^-^-regulated and NO_3_^-^/hormone co-regulated) and the comparison of their behavior allowed us to hypothesize how NO_3_^- ^and hormonal signaling pathways are connected in gene networks.

Despite these striking examples, very little is known concerning the transcriptional gene regulatory networks involved in NO_3_^- ^/hormonal cross-talk. Here, as a step towards understanding such transcriptional co-control, we present a computational biology approach (Figure 1) designed to discover genes that are regulated in response to nitrate treatments across a range of background conditions. Further analysis identified a refined subset of gene clusters to be under the control of both NO_3_^- ^and hormone signaling. We found that genes controlled both by hormones and NO_3_^- ^are more responsive to NO_3_^- ^and/or have a significantly higher level of baseline expression, than genes controlled by NO_3_^- ^alone. This analysis lead us to generate a new hypothesis that hormone signals play a role in enhancing the effects of the NO_3_^- ^signal. Moreover, we identified cis-regulatory elements (CREs) in the promoters of these genes that are candidates for enhancing the nitrate-regulation of gene expression.

## Results

### Bicluster analysis identifies a nitrate-responsive "biomodule"

In a previous study, meta-analysis of transcriptomes of NO_3_^- ^treated plants revealed that gene responses to nitrogen were very context-dependent, and only a very small number of core genes are regulated by NO_3_^- ^in a context-independent manner [2]. The underlying rules of such coordination/context dependence between signals had recently been proposed at a genome wide level concerning the interaction of carbon, nitrate, and light [3]. Moreover, in light of the context dependent nature of the N-response, mono-dimension clustering algorithms will miss genes that are co-regulated by N across a subset of treatment conditions. By contrast, an approach also known for decades [24] called biclustering can be used to identify nitrate responsive genes that are co-regulated, as a group, in response to a *subset *of nitrogen treatments across a matrix of meta-data (Figure 1) [25], likely susceptible to tackle the context dependence response to NO_3_^-^. Thus, detected biclusters are subsets of the studied genes exhibiting consistent patterns over a subset of N-treatment conditions. Such sets of genes would not be found using mono-dimension clustering approaches, which require that the genes in the cluster behave the same across *all *treatments. We used this biclustering method to analyze five microarray data sets from N-treatments of Arabidopsis generated by three different laboratory groups: the Crawford lab [26,27] (16 Affymetrix chips including controls), the Stitt lab [28] (14 Affymetrix chips including controls) and the Coruzzi lab [29,30] (46 Affymetrix chips including controls). This combined meta-data set resulting from N-treatments corresponded to a total of 76 microarray chips with controls [For details see Additional File 1].

**Figure 2:**
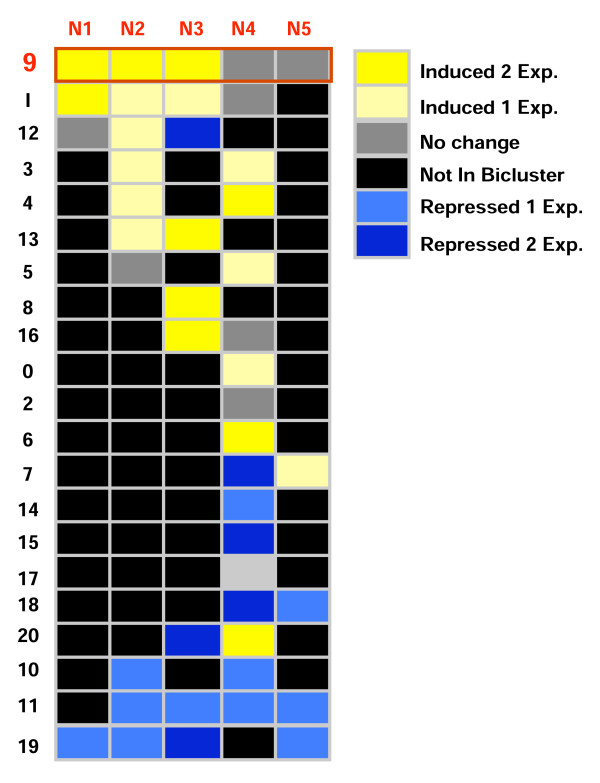
**Identification of 21 N-responsive biclusters: Nitrogen-responsive genes sets identified across subsets of N-microarray meta-data**. Heatmap of the responsive conditions of N-biclusters. The colors in the heatmap represent the kind of regulation (induced/depressed) and the degree of reproducibility for each data set captured in the N-biclusters. N1 to N5 represent the data sets from 3 different labs. N1:(Wang et al., 2003); N2: (Wang et al., 2004); N3: (Scheible et al., 2004); N4: (Palenchar et al., 2004); N5 (Gutierrez et al., 2007). The details of the experimental conditions of these data set is described in Additional File 1. N-bicluster 9 had the greatest degree of reproducibility over the greatest number of experiments.

To identify N-regulated genes in this meta-dataset, we first used a filtering step that consisted of selecting genes that are significantly regulated by at least one N-treatment across the various data sets (for filtering conditions see Methods, Figure 1). We found 3,782 such genes under the control of nitrogen in at least one of the considered N treatment conditions. This gene list was then used to generate 21 biclusters (containing 1,124 genes) using the SAMBA algorithm as implemented in the Click and Expander software package [31,32] (Figure 2). In order to identify whether the N-regulated biclusters had biological significance, we used the BioMaps analytical tool [30] to determine significantly over-represented functional categories using the MIPS annotation [33] in each of the 21 N-biclusters generated [Additional File 1]. Significant over-representation was determined based on a p-value that passed a 5% False Discovery Rate (FDR) cutoff (see Methods). Of the 21 N-responsive biclusters, we selected the bicluster # 9 (N-bicluster 9) for further investigation, based on the following criteria: i) genes in bicluster 9 show a reproducible response to N-treatment across several microarray datasets from several different studies (Figure 2), ii) the annotation of genes in N-bicluster 9 suggests that the genes in this bicluster have an important biological function since it comprises genes from *all *the known steps of N-uptake and N-assimilation (Table 1) [N-bicluster 9 gene list is provided in Additional file 1], and iii) N-bicluster 9 contained significant overrepresentation of MIPS functional categories spanning Amino Acid Metabolism, Carbohydrate Metabolism, and Transported Compounds, suggesting that the underlying regulatory mechanism serves to coordinate systems-wide responses to N (see Table 1). Here, it is noteworthy that this bicluster gathers relevant gene functions. As a matter of fact this coordination of a group of gene involved in the same functions can be designated as a "biomodule" [34-36]. Thus in this context, we used this terminology in the rest of our work.

**Table 1:** Over-represented functional categories in N-bicluster 9

**Term**	**Observed Frequency %**	**Expected Frequency %**	**P-value**
**ENERGY**	14.30%	1.50%	1.99E-11
pentose-phosphate pathway	4.80%	0.10%	3.13E-08
metabolism of energy reserves (e.g. glycogen, trehalose)	3.20%	0.10%	0.00073
glycolysis and gluconeogenesis	5.60%	0.60%	0.00027
**METABOLISM**	21.40%	6.50%	9.80E-07
C-compound and carbohydrate metabolism	13.50%	2.80%	2.62E-06
amino acid metabolism	6.30%	0.90%	0.00059
assimilation of ammonia, metabolism of the glutamate group	4.00%	0.20%	5.35E-05
nitrogen and sulfur metabolism	4.80%	0.30%	4.35E-05
**TRANSPORTED COMPOUNDS (SUBSTRATES)**	5.60%	1.10%	0.0161

This table indicates the functional terms that are over-represented in N-bicluster 9 vs. the whole genome using BioMaps with a 5% FDR cutoff to determine the statistical significance of p-values (see Methods for approach). The frequency of these terms in the functional annotation of the genes from N-bicluster and the p-value generated by BioMaps are indicated.

**Figure 3:**
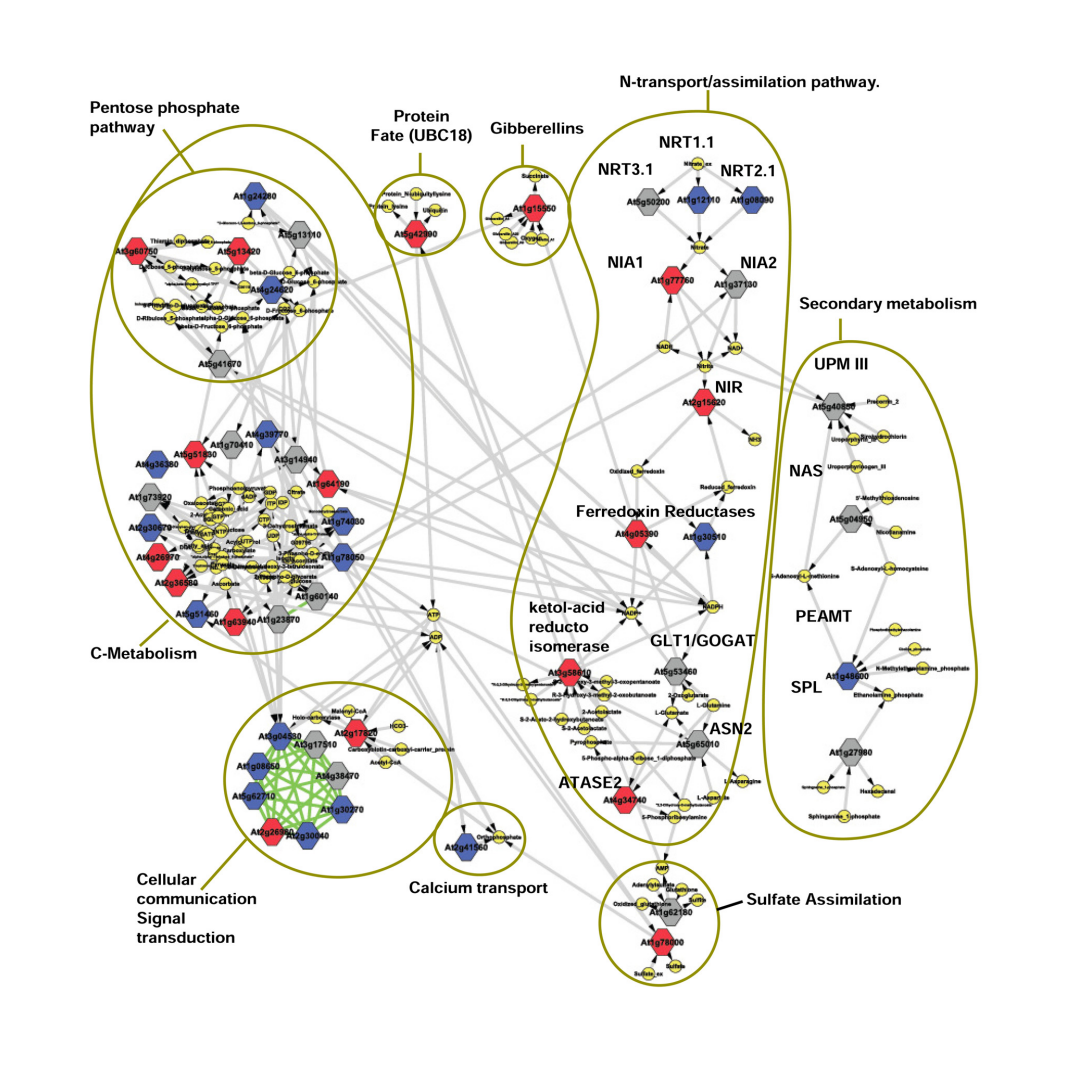
**N-bicluster 9 gene network: A highly connected network of biologically related gene functions**. The metabolic layer of information about gene connectivity in the Arabidopsis Multinetwork [30] was queried with the 126 genes belonging to the N-bicluster 9. Nodes represent genes (colored squares) and metabolites (yellow circles) connected by edges (metabolic interactions are colored in grey, protein:protein interactions are colored in green). Genes belonging to: i) N-bicluster NO_3_^- ^exclusive genes are colored in blue, ii) Significant N/H-biclusters are colored in red, iii) Other N/H-bicluster are colored in grey.

To determine the connectivity between the genes in N-bicluster 9, we queried the *Arabidopsis *multinetwork as described in [30] using the Virtual Plant software package . The *Arabidopsis *multinetwork is a model that integrates information for gene interactions based on a variety of data including: *Arabidopsis *metabolic pathways, known protein:protein, protein:DNA, miRNA:RNA interactions, and predicted protein:protein and protein:DNA interactions [30]. From the network analysis of 126 genes in N-bicluster 9, a network comprised of information from metabolic and protein:protein connections included 179 nodes (52 genes and 127 metabolites) linked by 261 functional relationships (Figure 3). To test whether this level of connectivity was greater than expected by chance, we computed a p-value by comparing this network to networks generated from a collection of genes of the same size, randomly sampled from the 22,746 genes present on the full genome chip (p-value < 0.0001), computed as described in [37]. Thus, this analysis confirms that the 126 genes in N-bicluster 9 display a significant level of connectivity, providing added support that the biclustering approach has identified a biologically functional regulatory module or "biomodule" [34-36].

### The genes in the N-bicluster 9 "biomodule" are induced by nitrate as a signal

**Figure 4:**
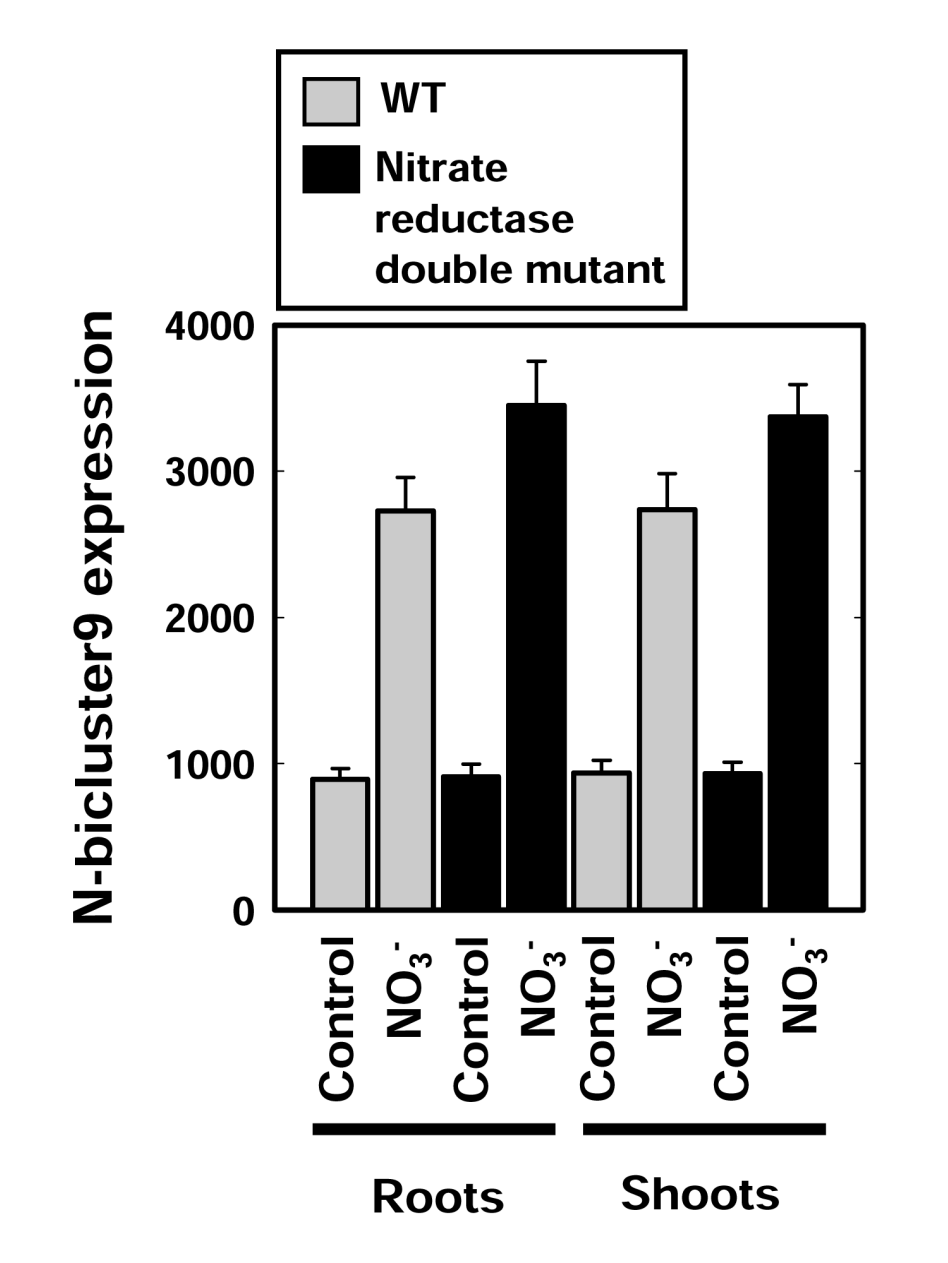
**The 126 genes in N-bicluster 9 are NO_3_^- ^regulated**. Average raw MAS5 processed signal values for the 126 genes from N-bicluster 9 in the nitrate reductase double mutant data [27]. Values in these plots indicate the average expression for each condition taken from the nitrate reductase double mutant data set where plants were treated with KNO_3 _or an equal concentration of KCl (Control).

Here, we asked which N-signals were involved in mediating the regulation of genes in N-the bicluster 9 biomodule. Nitrogen-responsive genes are known to be regulated by various endogenous and external nitrogen signals. For example, nitrate itself as been identified as a signal that regulates a large number of genes genome-wide [27]. In specific examples, nitrate can act as an inducer *and *a repressor of *NRT2.1*, which codes for a major component of the nitrate high affinity transport system [21,22,38]. Moreover, N-reduced metabolites such as NH_4_^+^, and organic forms of nitrogen (glutamine or glutamate) have been shown to also control gene expression, again at a genome-wide level [39,40], which was also demonstrated in specific examples [38,41]. Because NO_3_^- ^is quickly assimilated into N-reduced and organic-N compounds, we tried to elucidate which form of nitrogen regulates the expression of genes in N-bicluster 9. For this, we mined Affymetrix microarray data obtained from nitrate treatments of a nitrate-reductase T-DNA double mutant knockout from Wang et al [27], which was not included in the biclustering data set. This microarray data set was derived from wild-type (WT) and nitrate reductase (NR) null mutant *Arabidopsis *plants treated with KNO_3 _for 2 hrs. The NR double mutant is a T-DNA knockout mutant that is deficient in both structural genes encoding nitrate reductase (*nia1 *and *nia2*) and is therefore unable to reduce nitrate to downstream forms of nitrogen [27]. Thus, any genes that respond to nitrate in wild type and the NR double mutant are likely to be controlled by nitrate itself. We found that genes in the N-bicluster 9 biomodule are regulated in response to nitrate, since their nitrate responsiveness is preserved in the *nia1*/*nia2 *NR double mutant at levels identical to wild-type (Figure 4).

### Identification of nitrate/hormone responsive biclusters

**Figure 5:**
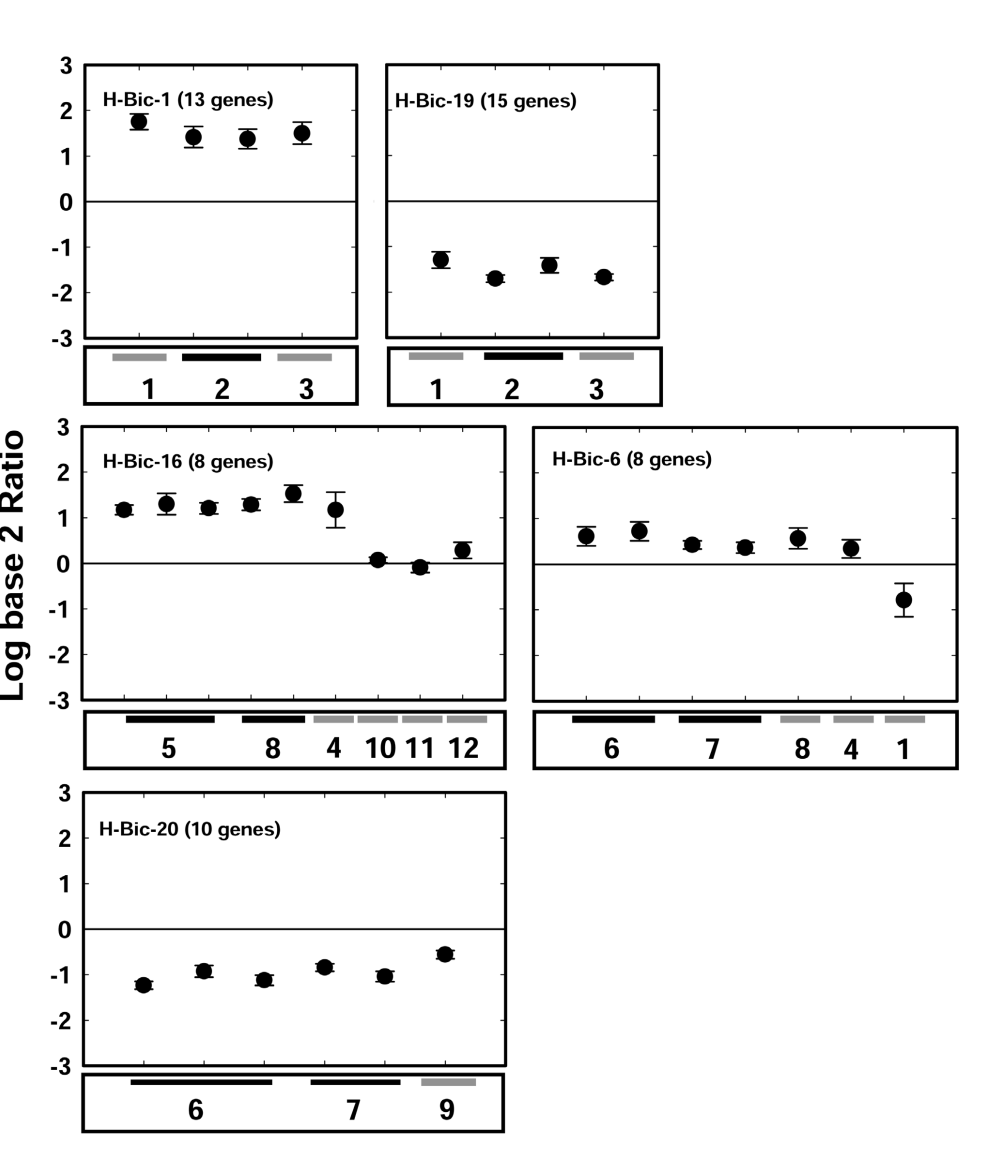
**Hormone responsiveness of the five selected N/H-biclusters from N-bicluster 9**. Centroid plots of the expression patterns for genes across the 5 significant (> 50% reproducibility; > 1.5 fold change) N/H-biclusters. Black bars below the plots indicate replicate experiments and grey bars indicate singleton experiments. N/H-biclusters 1 and 19 show a strong response to ABA treatment during seed imbibition (induction and repression respectively), N/H-biclusters 16 and 6 show an induction to cytokinin in roots and shoots of mutant and wildtype plants respectively. N/H-bicluster 20 shows a strong depression due to cytokinin in root tissues of mutant and wild type plants. Treatments are taken from the NASC data set: 1- Brassinolide 10 nM 3 hours (Seedlings), 2- ABA 3 μM 24 H (during Seed Imbibition), 3- ABA 30 μM 24 H (during Seed Imbibition), 4- Zeatin 20 μM 1 H (Seedlings), 5- Zeatin 20 μM 1 H (Shoots), 6- Zeatin 20 μM 1 H (Roots), 7- *arr10/12 *Zeatin 20 μM 1 H (Roots), 8- *arr10/12 *Zeatin 20 μM 1 H (Shoots), 9- AtIPT8/pga22 (Seedlings), 10- *ga1–5 *GA3 1 μM 0.5 H (Seedlings), 11- IAA 1 μM 0.5 H (Seedlings), 12- IAA 1 μM 1 H (Seedlings).

As stated above, the N-regulation of the bicluster 9 "biomodule" appears to have biological relevance as it contains genes covering all steps in the N-uptake/assimilation pathway, and genes integrating the regulation of genes in N-metabolism with other related metabolic processes including C-metabolism and Energy. As many of the genes in these pathways have been shown to be under control of other hormones [7,42,43], we hypothesized that hormonal control might play a role in the regulation of the genes in the N-bicluster 9 biomodule. To test this hypothesis, and to uncover the possible mechanistic basis for nitrate/hormome signaling interactions, we performed an *in silico *analysis of N-bicluster 9 gene regulation in response to hormones. For this analysis, we constructed a meta-data matrix of microarray experiments from the NASC repository (Nottingham *Arabidospsis *Stock Center, [44]) covering all available hormone and hormone inhibitor treatment experiments (including auxin, abscisic acid, ethylene, cytokinin, brassinosteroids, giberellic acid and jasmonic acid). We included all data sets having replicates and a relative control (see Methods). The data were converted to a log base 2 ratio of treatment/control, and biclustering was used to identify sets of genes co-regulated under various hormone treatments. Biclustering of the 126 genes in the N-bicluster 9 biomodule over the hormone meta-data set generated 22 hormone biclusters referred to as N/H-biclusters (Nitrogen/Hormone responsive). These N/H biclusters encompass 77 genes contained within N-bicluster 9 [see Additional File 1 for a complete list of N/H-biclusters]. Each of these N/H biclusters has an average number of 8 genes with extensive overlap between several N/H biclusters. These results show that 77 of the genes (out of 126) in N-bicluster 9 are reproducibly regulated by both nitrate and hormone treatments. Further the fact that these genes overlapped between N/H biclusters suggested that they may be responsive to several different types of hormone treatments. The 22 N/H-biclusters were classified according to filtering criteria described in Methods section (Table 2). Out of the 22 N/H-biclusters, five biclusters which showed the most extensive and reproducible responses to hormones were selected for further analysis (Figure 5). The metabolic and functionally interacting genes contained in these N/Hormone biclusters are highlighted in the 77 N/H responsive genes in the network view of the N-bicluster 9, as depicted in Figure 3.

**Table 2:** NO_3_^-^/Hormone Response Interaction.

**Description of Comparison**	**Coefficient Estimate**	**P-value**
**N/H-bicluster 1 vs. Exclusive +N/H-bicluster 6**	1721.4	4.08E-05
**N/H-Bicluster 16, 19, 20 vs. Exclusive + N/H-bicluster 6**	761.8	0.0099
**Treated vs. Control**	1290.1	5.82E-07
**N/H-bicluster: Treated**	2190.2	0.00022
**N/H-Bicluster 16, 19, 20: Treated**	1011.9	0.01539

Analysis of variance that examines the response to nitrate treatment of N-bicluster 9 genes that fell into various hormone biclusters or failed to appear in a hormone bicluster. Coefficients and p-values in the simplest model that retained explanatory power. The gene-expression response variable is determined by main effects of *Treatment *(with 2 levels: control and treated), *N/H bicluster *(with 3 compound levels), and an interaction term between *N/H bicluster *and *Treatment*. In this model, N-bicluster 9 exclusive genes and N/H bicluster 6 genes form an aggregate "control" group with no significant difference between them. A second, significantly different, aggregate group is comprised of genes in N/H-biclusters 16,19 and 20. A third group includes genes from N/H-bicluster 1 only. The control level for the *Treatment *factor is KCl. In this table each main-effect coefficient represents a difference in expression from the group comprised of N-bicluster 9 exclusive and N/H bicluster 6 genes under KCl treatment. The interaction term is over and above the main effects. For example, the difference between the mean expression of N/H-bicluster 1 genes under nitrate treatment and the mean expression of the control-group genes under control treatment (KCl) is obtained by summing: the main effect of N/H-bicluster 1 (1721.4); the main effect of (nitrate) Treated (761.8); and the term representing the interaction between N/H-bicluster 1 and Treated (2190.2).

The analysis of the N/Hormone biclusters (biclusters 1, 6, 16, 19, 20) revealed that cytokinin and ABA are the main hormone treatments under which the NO_3_^- ^regulated genes from N-bicluster 9 are co-regulated (Figure 3 and 5). N/H-biclusters 1 and 19 are both mainly driven by ABA treatments, although their respective regulation is in opposing directions (induced vs. repressed). N/H-biclusters 6, 16, and 20 are comprised of genes almost exclusively regulated in response to cytokinin treatment (Figure 5). Together, our results suggest that the coordinated regulation of these genes to nitrate as well as cytokinin or ABA may be part of a regulatory network that mediates the responsiveness of these genes.

### Functional interactions within the nitrate/hormone biclusters

As all N/H-biclusters were derived from the 126 genes contained in N-bicluster 9, we used a modified version of BioMaps analysis to determine which if any of the five selected N/H-biclusters were enriched for specific MIPS functional categories (see Methods). This analysis demonstrated that N/H-biclusters 1, 16 and 19 had at least one over-represented MIPS category, when compared to N-bicluster 9 [see Additional File 1]. The most significantly over-represented categories from these N/H-biclusters are genes involved in metabolic pathways, suggesting that this NO_3_^-^/Hormone "crosstalk" may be directed towards the coordinate regulation of genes in interconnected metabolic pathways (see Network View of N-bicluster 9). Further, genes from N/H-bicluster 1 have several additional categories over-represented including Energy, Pentose phosphate pathway and Photosynthesis.

### Elucidation of a hormone "enhancement" of gene nitrate responsiveness

**Figure 6:**
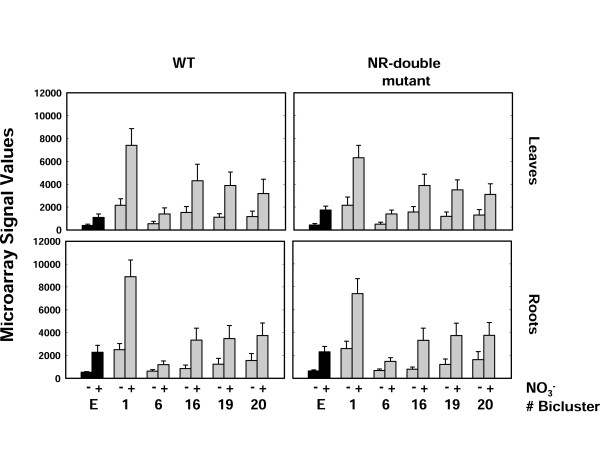
**Changes in NO_3_^- ^responsiveness for genes in significant N/H biclusters vs. N-bicluster 9 exclusive genes**. Histogram plots of average expression over NO_3_^- ^treatment conditions in both WT and NR double mutant microarray data showing the nitrate responsiveness of genes that were present in significant hormone biclusters and genes that were not present in any N/H-bicluster (N-bicluster 9 exclusive (E)), data indicates root and shoot responses in WT and NR double mutant plants). Comparison between treatment and control across the different groups indicates a clear difference in both the baseline expression level and average response to nitrate for N/H-biclusters 1, 16, 19 and 20 and was confirmed with ANOVA (see text). (+) KNO_3 _treatment, (-) KCl treatment.

With the aim of elucidating the mechanisms which mediate differences in regulation between genes that are controlled by NO_3_^- ^only *vs*. genes that are co-regulated by NO_3_^- ^and hormones, we analyzed the expression of the genes N-bicluster 9 and N/H bicluster in the NR double mutant data set [27]. This analysis uncovered a strong and unexpected difference between: a) the NO_3_^- ^responsiveness of genes in all of the five N/H-biclusters *vs*. b) genes from N-bicluster 9 that *did not *appear in any hormone bicluster (termed "N-bicluster 9 exclusive") (Figure 6) (see also informatic analysis scheme Figure 1B). Based on the analysis described below, genes belonging to N/H-biclusters 1, 16, 19 and 20 are significantly more NO_3_^- ^responsive as measured by the amplitude of expression in genes from N-bicluster 9 that do not fall into any N/H-bicluster (N-bicluster 9 exclusive genes) (see results of ANOVA, Table 2). Further, we validated that the enhanced nitrate responsiveness for the genes belonging to the N/H biclusters, is exhibited not only in the NR double mutant data set, but also in the entire N-treatment meta-data set used to build the N-biclusters [N1 to N5, see Additional File 1] (data not shown).

To quantify and statistically validate the regulation and nitrate-responsiveness of the genes in the N/H biclusters in the NR double mutant data set (Figure 6), we modeled the expression of genes from these groups using the *lm*, *summary.lm *and ANOVA functions in [R] [45]. In this analysis the gene-expression response variable was modeled as a function of 4 explanatory-variable factors: i) *Treatment*, with 2 levels (nitrate treatment and control); ii) *Tissue*, with 2 levels (roots and shoots); iii) *Genotype*, with 2 levels (mutant (NR double mutant) and wild-type); iv) *N/H-bicluster*, with 6 levels (N/H bicluster 1, 6, 16, 19, 20 and N-bicluster 9 exclusive). To avoid any ambiguity between factor levels, overlapping genes from H-biclusters were removed from the analysis. The response variable (signal values) were taken from the normalized MAS5 data from the Wang *et al*, dataset [27] (These data were used to build Figure 6). In our ANOVA analysis, we started with an initial model that included main effects for each of the factors and an interaction term for the *Treatment *and *N/H-bicluster *factors. We simplified the model systematically in a step-wise procedure as outlined in Crawley [46] and fully described in Additional File 2. Briefly, our results from ANOVA analysis showed that the main effects of *Tissue *and *Genotype *factors were not significant (p-values of 0.13 and 0.94, respectively). Further it revealed that the factor levels of *N/H-biclusters *were not all significantly different from each other. Specifically NH-bicluster 16 19, and 20, in one hand, and 6 and N-bicluster 9 exclusive, in the other hand, were not significantly different from each other so that these levels could be combined into a single compound level. The final result of our simplification procedure was a model with main effects of *Treatment *(with 2 levels), *N/H bicluster *(with 3 compound levels), and an interaction term between *N/H bicluster *and *Treatment*. The R code and output for this model simplification are fully available in Additional File 2.

Using this final model, we were able to show that the N/H bicluster 1 level and the compound level for N/H biclusters 16, 19 and 20 are both significantly different from the compound level of N/H bicluster 6 and N-bicluster 9 exclusive in both having a stronger baseline response and a stronger response to nitrate (see Figure 6). Taken together our combined bicluster analysis data from N-treatment and hormone-treatment meta-datasets, leads us to propose a new hypothesis that hormone signaling (specifically ABA, and/or cytokinins which are represented in N/H biclusters 1, 16, 19 and 20) may act as an enhancer of NO_3_^- ^signaling/induction.

### Identification of candidate cis-binding elements involved in the hormonal enhancing effect of the nitrate response and *in silico *validation

In order to determine candidate cis-regulatory elements (CREs) involved in the proposed 'enhancing' effect of hormones on nitrate signaling, we first scanned the ~3,000 bp parsed upstream promoter sequence of the genes in N-bicluster 9 for known transcription factor binding sites using a DNA pattern search tool [47]. We next determined the over-representation of these CREs in the 126 N-bicluster 9 genes using the Fisher Exact Test (see Methods). Based on this analysis, 23 CRE elements were found to be significantly over-represented in N-bicluster 9, compared to the genome-wide frequency of these elements. These CREs could potentially be involved in nitrate responses, or in a yet to be defined signal (or complex of signals) controlling this gene cluster as a whole (Table 3). Using a similar approach we tested for a significant difference in the CRE frequency for these CREs between two groups: one containing the N/H bicluster 1, 16, 19 and 20 genes and the other containing the N-bicluster 9 exclusive (background). N/H bicluster 6 was removed from this and all subsequent analysis as it was shown to not be significant in the previous ANOVA and contained very few genes (2 genes) when overlapping genes were removed. This analysis revealed that 2 CREs, HSE element [48] and E2F element [49]. These two CRE elements are significantly over-represented in the N/H biclusters (See Table 4) analyzed as a group compared to their frequency within the subset of N-bicluster 9 genes not present in any of the N/H-biclusters (termed NO_3_^- ^exclusive). The specific over-representation of these CREs in the N/H biclusters implicates them as potential candidates to be involved in the cross-talk between hormone and NO_3_^- ^response. The known physiological roles of the E2F and HSE CREs are discussed below.

**Table 3:** Over-represented known CREs in N-bicluster 9.

**Cis Regulatory Element**	**% Present in Whole Genome**	**% Present in N-bicluster 9**	**P-value**
BoxII	49.32%	66.67%	6.90E-05
RAV1A	84.00%	94.44%	1.94E-04
Wbox	80.83%	92.06%	3.40E-04
MYB2CONSENSUSAT	63.96%	78.57%	3.43E-04
RY_repeat	5.36%	13.49%	4.09E-04
ebox	86.24%	95.24%	5.87E-04
DPBF1&2_binding_site_motif	64.32%	77.78%	9.66E-04
ARF	45.03%	59.52%	0.001096
AtMYC2_BS_in_RD22	44.10%	57.94%	0.001509
MYB4	72.72%	84.13%	0.001727
Bellringer/replumless/pennywise BS1 IN AG	42.04%	55.56%	0.001932
SV40	25.69%	38.10%	0.001954
			
ATB2/AtbZIP53/AtbZIP44/GBF5BS in ProDH	48.52%	61.90%	0.002188
CCA1	34.48%	47.62%	0.002400
			
ABRE like	26.46%	38.10%	0.004204
AtMYB2 BS in RD22	16.57%	26.19%	0.005280
T box	58.94%	70.63%	0.006024
LFY	62.88%	73.81%	0.007078

Results of a promoter sequence analysis of genes from N-bicluster 9 with the Fisher Exact Test used to determine CRE frequency over-representation in N-bicluster 9. Over-representation of CREs was determined by comparing the frequency of these sites in N-bicluster 9 genes to the frequency in the whole genome as a background. Significance was determined using a 5% FDR cut-off. Indicated in this table is the percentage of genes from the whole genome and N-bicluster 9 that contain these elements and the respective p-values for over-representation. *P-values 0.01–0.05; **P-values 0.001–0.01; ***P-values < 0.001.

**Table 4:** Over-represented CREs for 4 significant N/H-biclusters.

**CRE Name**	**P-value (N/H-Bicluster vs. N-bicluster 9 Exclusive)**	**% Present in N/H-bicluster Genes**	**% Present in N-bicluster 9 Exclusive Genes**	**% Present in N-bicluster 9 Genes**
**E2F**	0.0022**	60.5%	27.08%	40.48%
**HSE**	0.0089**	60.5%	31.25%	42.06%

Results of a promoter sequence analysis of genes from 4 significant N/H biclusters based on ANOVA with the Fisher Exact Test used to determine CRE frequency over-representation of in the collective gene list (38 genes) *vs*. N-bicluster 9 exclusive genes. Indicated in this table are the p-values for over-representation, the frequency of these CREs in the 4 N/H biclusters, the frequency of the same CREs in the N-bicluster 9 exclusive gene list and the frequency of these CREs in the full N-bicluster 9 gene list. Based on the analysis of the 4 significant N/H-biclusters, only the p-values for the HSE and E2F CREs were significant, indicating over-representation of these CREs (significance based on a 5% FDR cutoff). **P-values 0.001–0.01

## Discussion

### Biclustering identifies a "biomodule" of biologically related nitrate-regulated genes involved in metabolism and signal transduction

In a previous meta-analysis of nitrate-regulated genes, we demonstrated that a very small number of genes are nitrate regulated across a variety of background conditions, while the vast number of nitrate-regulated genes are regulated in a context-dependant manner [2]. This observation suggests that the NO_3_^- ^signaling pathway is also under the influence of other (as yet) unidentified controls. Taking this observation as a starting point, we decided to use biclustering technique: an approach that clusters both genes *and *treatments, as a tool to discover genes that are co-regulated by nitrate across a wide variety of background conditions corresponding to a subset of the meta-data analysis. This biclustering approach allowed us to uncover a "biomodule" of 126 NO_3_^- ^regulated genes that are related in expression pattern and in biological function (N-bicluster 9, Figures 2 and 3, Additional file 1, table s2). Indeed, as a group, the genes in N-bicluster 9 comprise a set of 52 metabolic genes including, for example, all steps in the pathway of nitrate uptake & reduction (*NRT1.1, NRT2.1, NRT3.1, NIA1, NIA2*, NIR), as well as genes involved in N-assimilation into organic form (*GDH1*, *ASN2 *and *GLT1*). In addition, the N-bicluster 9 also contains significant overrepresentation of genes involved in Energy, Nitrogen and Carbon metabolism (Table 2). This strong functional coherence of the genes in N-bicluster 9 is illustrated by the interactions between 52 genes in the metabolic/protein interactions shown in the subnetwork (Figure 3).

It is noteworthy that the concept of the 126 genes in bicluster 9 constituting a "biomodule" in our study is comparable to ideas that have been already developed by others in the field of systems biology. For instance, i) Baliga et al. [50] state that "a biomodule is a group of proteins that execute a particular function", and ii) Bonneau et al. [51] also used a biclustering approach (cMonkey) to define "biologically meaningful biclusters". The conjunction of both above definitions match our concept/definition of a "biomodule".

As an insight into potential TFs that regulate the genes in this network, it is noteworthy that N-regulated bicluster 9 contains 17 transcription factors (based on AGRIS transcription factor annotation) whose regulation is by definition correlated with targets in N-bicluster 9, as well as with genes from other functional and unknown categories. N-bicluster 9 also contains other regulatory genes potentially involved in signal transduction such as kinases or phosphatases (6% of the genes from this N-bicluster fall into this category) (Table 1).

### Hormones enhance the NO_3_^- ^responsiveness of genes within the bicluster 9 biomodule: what are the physiological consequences?

To identify potential regulatory mechanisms for the NO_3_^- ^responses of these genes to by other stimuli, we examined the regulation of the 126 genes in N-bicluster 9 across a metadata set of hormone microarrays. This was done in order to try to understand whether these genes, or subsets of these genes, are coregulated by hormones as well. Hormone treatments have been previously shown to have strong interactions with nitrogen signaling [5]. This analysis identified a subset of 77 genes in N-bicluster 9 that also cluster together across a subset of hormone treatments. The position of these 77 genes present in the N/Hormone biclusters are shown in the context of the metabolic/protein interaction network presented in Figure 3. This view demonstrates a strong potential effect of diverse hormonal controls on the level of response of NO_3_^-^-controlled metabolic processes [See Figure 3 color coding for nodes: Red squares = significant N/H-bicluster genes (genes from N/H-biclusters 1, 16, 19 and 20), Blue squares = genes controlled only by NO_3_^- ^and not co-regulated by hormone treatments, based on results of hormone biclustering, Grey squares = non-significant N/H-bicluster genes controlled by hormones (i.e. no reproducible hormone response in N/H biclusters, see Methods)]. This hormonal control of nitrate-regulated genes represents a potential mechanism to fine tune and co-ordinate response levels of genes in a biomodule so that metabolic processes (here N-assimilation, carbon metabolism, and signaling components) can be regulated according to the growth rate of the plant. This is consistent with the observations made for phosphate [52,53], sugar [54], sulfate [55], and iron metabolism [56]. In all of these previous studies, when the hormone receptor is mutated, the response of genes to the nutrient under investigation is maintained, but the hormone response of the same genes is abrogated. This implies that hormonal control of nutrition pathways has a broad effect and controls metabolism as a whole, and is distinct from nutrient signaling. Our current work supports this view and also goes a step further. Indeed, our systems approach has enabled us to derive the hypothesis that hormone signals can interact with NO_3_^- ^signals to enhance the responsiveness of genes, and we have performed and *in silico *test of this hypothesis. This hypothesis is based on the finding that genes controlled by NO_3_^- ^only, were shown to be less responsive to NO_3_^- ^than genes under the control of NO_3_^- ^*and *hormones (Figure 6, Table 2). This kind of interaction has to our knowledge never been reported, and is a particularly novel aspect of for the effect of hormones as they relate to NO_3_^- ^induction. Although the effect of external hormone supply on genes belonging to NO_3_^- ^assimilation pathway or sensing system has already been documented, our results propose a new dimension of interaction at the transcriptional level between hormonal and NO_3_^- ^signaling. The existence of specific links between different nutrient and hormonal signals reported herein is also of particular interest and deserves further investigation.

### Putative roles of the E2F and HSE Cis Regulatory Elements (CRES) in mediating cross talk between nitrate and hormone signaling

Our study has identified two putative regulatory elements that are over-represented in the four significant N/H-biclusters identified by ANOVA (Table 2). To identify the potential role of such elements in mediating the hormone enhancing effect on nitrate responsiveness, we performed an *in silico *analysis aiming at deciphering the potential effect of each candidate binding site. By removing all genes from N-bicluster 9 exclusive gene list that contained these CREs (E2F and HSE), we were able to "virtually" examine their respective role in the enhancement of the baseline and NO_3_^- ^response by comparing these genes to genes from significant N/H-biclusters 1, 6, 16 and 20. The analysis demonstrated that E2F and HSE CREs are potentially involved in the hormonal enhancing effect of expression of these NO_3_^- ^responsive genes (Table 4, 5, and 6). To date, the heat shock elements (HSE) were not shown to be involved in the control of N-regulated genes though their role in *Arabidopsis *in the transcriptional control of responses to heat stress has been extensively studied [57]. However, a heat shock transcription factor *HsfA9 *has been shown to be under hormonal control in seeds (ABA through ABI3) [58]. This observation leads to the tentative hypothesis that heat shock elements could potentially be involved in conveying a hormonal signal. Moreover, to further have insight into the HSF/hormonal connection we ran a Sungear [59] analysis to decipher if these factors are under any other hormonal controls. To do so, we queried gene annotation for HSF term. We found 21 HSF, and looked to see if they were found regulated by any hormone as reported by Nemhauser et al. [60]. Out of the 21 HSF detected we found that 6 (28%) are regulated by ABA (2 of which are also regulated by methyl-jasmonate), and 1 gene is regulated by cytokinins. This kind of co-regulation might further support the potential connection between HSF and hormonal signals.

E2F binding elements and the role of their associated transcription factors are still poorly understood in plants. However, what is known in plants as well as in other organisms is that these factors (considered in animals as oncogenes) are involved in the control of the cell cycle [61,62]. Remarkably the role of E2F in the control of gene expression related to N-assimilation has already been shown in *Arabidopsis*, providing an independent validation of our results. Vlieghe et al. [63] demonstrated that the over-expression of the E2Fa-DPa transcription factor leads to the induction of nitrate reductase (*NIA2*), glutamine synthetase (*GS*), glutamate synthase (*GOGAT*), and nitrite reductase (*NIR*) gene. It is noteworthy that all of these genes respond to both nitrate and hormonal signals in our analysis (Figure 3). Furthermore, several genes in the N/H biclusters that are involved in C-metabolism are also mis-regulated in plants over-expressing E2Fa-DPa. Interestingly, E2F CREs were also identified in the nitrate reductase promoter of the green algae, *Chlorella vulgaris*. The protein binding activity at this site was validated but was not dependent on nitrate in the media [64]. This confirms the idea that E2F CREs are involved in the interaction of the NO_3_^- ^response with other signals such as hormones and may be mediating crosstalk between these signals. Finally, the cell cycle is known to be an important target of hormonal signaling. For instance the *Arabidopsis *E2FC-DPB transcription factor was demonstrated to be involved in the control of the cell cycle. Also, cell division (monitored by CYCB1-GUS) in plants over-expressing E2FC-DPB was found to be less sensitive to auxin than cell division in wild types plants. This supports the hypothesis that E2F transcription factors are involved in mediating hormonal control of cell division [65].

## Conclusion

In conclusion, our results suggest and highlight a significant level of control of NO_3_^- ^signaling by hormones. This control may allow plants to modulate biomodules of genes spanning N and C metabolism according to growth-dependant hormone signals. The systems biology approach presented herein demonstrates the inference of relationships between signals *a postriori *using extensive microarray data sets (76 chips for Nitrogen + 278 chips for hormones) to uncover new hypotheses for mechanisms underlying the much studied but poorly understood interactions between nutrient and hormone signaling. This *in silico *approach opens the door toward unraveling new biological concepts by systems analysis of existing microarray and other genome scale data sets within the public domain.

## Methods

### Nitrogen Microarray Meta-Data set used for bicluster analysis

Expression values for all genes within the *Arabidopsis *genome present on the Affymetrix chip were taken from published data on nitrogen treatments vs. controls for all the available experiments from the data sets published in:[26-28,30]. All microarray data used in this analysis was processed and normalized using Affymetrix Suite 5.0 or MAS5 Software (as implemented in the R statistical package [45] the two normalization Methods gave equivalent results. For biclustering analysis (see below), signal values were converted to log base 2 ratios with the treatment condition compared to its relative baseline condition (control). Genes with raw signal values less 100 in their treatment or control conditions in either replicate had their signal log ratio values replaced with a non-numerical NA value which is ignored by the biclustering algorithm. Finally, Log 2 ratio data from the microarray data was analyzed to determine which genes in the genome were greater then 1.5-fold responsive in any pair of replicate experiments in this meta data set. The resulting list of 3,752 N-responsive genes was used for biclustering, as described below.

### Hormone Microarray Meta Data Analysis

All hormone microarray data was taken from the MAS5-processed NASC Microarray database (Nottingham Arabidopsis Stock Center [44]) data. Data was chosen based on annotation and experimental conditions that referred to a hormone or hormone inhibitor treatment vs. a relative control, with only replicated data used for biclustering analysis. A total of 19 data sets comprising 278 microarray experiments were compiled based on these criteria. The full list of data sets and the contributing number of microarrays from each data set is provided in Additional File 1. All hormone data with signal values of < 100 had their signal values replaced with a non-numerical NA value. Further, all data was converted to log base 2 ratios prior to biclustering analysis. The 126 genes from N-bicluster 9 were biclustered over all hormone data as described (see below).

### Biclustering of Microarray Data

Biclustering was performed using the SAMBA algorithm as described by [32] and as implemented in the CLICK and EXPANDER program [31]. Biclustering was performed using default parameters except as follows: amount of overlap allowed (50%), gene coverage (set to cover all genes) and the number of genes expected (set to maximum number of genes in the data set). Biclusters that were used for further analysis were chosen based on genes being ≥ 1.5 fold regulated across reproducible experiments and the presence of replicate experiments for ≥ 50% of the experiments.

### BioMaps Analysis of N-biclusters

BioMaps analysis of N-biclusters was performed as described in [30] as accessed via . The program was run using the MIPS [33] annotation option for functional definitions. A 5% FDR (False Discovery Rate) cut-off was computed using the R statistical package [45] to determine significant p-values.

### Modified BioMaps of N/Hormone Biclusters

As N/H-biclusters were derived from N-bicluster 9, over-representation of MIPS [33] functional terms were determined using the Fisher's Exact Test as implemented in the R statistical package [45] to compare the proportions of genes from N/H-biclusters containing a MIPS term vs. the proportion of genes from N-bicluster 9 not in N/H biclusters containing that same term.

### Multinetwork Analysis of Bicluster 9

To understand the relationships among the 126 genes from N-bicluster 9, the *Arabidopsis *multinetwork analysis tool was used [30] as accessed by . This network contains many validated connections for gene interactions in the *Arabidopsis *genome. Network interactions were visualized using Cytoscape [66].

### Computing the False Discovery Rate (FDR)

An FDR control method was used to determine a significance cutoff for p-values. This value was computed using a script written for the R statistical package [45]. This script was derived from the Storey and Tibshirani method [67] which determines a cut-off based on the expected proportion of false positives incurred when calling a feature significant.

### Cis Regulatory Element (CRE) Detection Using Known CREs

In order to detect known CREs that may be over-represented in a group of genes (e.g. N-bicluster 9, N/H-biclusters), sequence analysis of the promoter regions of these genes was performed. We used *Cis *Regulatory Element (CRE) annotation from the AGRIS Database (Arabidopsis Gene Regulatory Information Server [68]) as well as our own literature search to identify biologically active CREs that have been validated by *in vivo *experimentation. CRE detection was performed using the DNA pattern search tool available from RSA Tools [47] upon 3,000 bp of parsed upstream promoter region (taken from the AGRIS database).

The test for over-representation of CREs was performed using Fisher's Exact Test. This test compared the proportion of promoters in which a particular CRE of interest appeared in one group with the proportion of these same CREs detected in another group. A p-value cutoff was computed using a 5% FDR cut-off for significance.

## Authors' contributions

GC, GK, DT and DN designed the study. DT and DN performed statistical analysis. GK and DN wrote the paper. All authors read and approved the final version of the manuscript.

## Supplementary Material

Additional file 1 **Additional Tables and Legends**. Contains all Additional Table and legend cited above including: 1) Summary Table of C/N Datasets Used to Generate N-biclusters; 2) BioMaps Analysis of N-biclusters; 3) N-bicluster 9 gene list; 4) Results of N/H Biclustering; 5) Significant H-biclusters Over-represented Functional Categories; 6) Hormone Microarray Datasets. Click here for file

Additional file 2 **ANOVA [R] script and results**. Contains the entire commented procedure of the ANOVA analysis, comparing N/H controlled genes *vs*. N-exclusive genes. Click here for file
