# Supplementary material for: A system biology approach highlights a hormonal enhancer effect on regulation of genes in a nitrate responsive "biomodule"
Source: BMC Syst Biol. 2009 Jun 6;3:59. doi: 10.1186/1752-0509-3-59 (PMC2702358; doi:10.1186/1752-0509-3-59)
Supplement: Additional file 1 — Additional Tables and Legends. Contains all Additional Table and legend cited above including: 1) Summary Table of C/N Datasets Used to Generate N-biclusters; 2) BioMaps Analysis of N-biclusters; 3) N-bicluster 9 gene list; 4) Results of N/H Biclustering; 5) Significant H-biclusters Over-represented Functional Categories; 6) Hormone Microarray Datasets. [file 1752-0509-3-59-S1.pdf]

Additional Table 1: Summary Table of C/N Datasets Used to Generate N-biclusters. Growth and treatment conditions used in the C/N dataset to generate nitrogen responsive biclusters. The columns correspond to the lab from which the data originated, the identification given to that dataset for this analysis, the pretreatment conditions the plants in these studies were grown on (Carbon/Nitrogen conditions), the type of treatment as well as the time and organ of treatment and the reference for each dataset.

Lab	ID	# of Chips	Pretreat.	Treatment						Reference
			Carbon/ Nitrogen	Carbon	Nitrogen	Time (HRs)	Organ			
							Roots	Shoots	Seedlings	
Crawford	N1	8	High NH_4-succ/ Low Sucrose		Low KNO3	0.3	+	+	-	Wang et al 2003
Crawford	N2	8	High NH_4-succ/ Low Sucrose		High KNO_3	2	+	+	-	Wang et al 2004
Stitt	N3	14	High NH_4-succ/ Low Sucrose	High Sucrose	High KNO_3	0.5; 3	-	-	+	Scheible et al 2005
Coruzzi	N4	34	Low KNO_3	High Sucrose	High KNO_3	8	+	-	-	Gutierrez et al 2007
Coruzzi	N5	12	Low KNO_3 Low NH_4NO_3 High Sucrose	High Sucrose	High KNO_3, NH_4NO_3	8	-	-	+	Palenchar et al 2004

Additional Table 2: BioMaps Analysis of N-biclusters. This table indicates the top over-represented category based on BioMaps analysis of N-biclusters. Top categories were those categories with the lowest p-value. The columns represent the individual N-bicluster number, the category name and the p-value for that category

Bic #	Top Overrepresented Categories in Biclusters	p-value
0	Cell Wall	0.00098
1	Energy	3.77E-05
2	Biosynthesis of Phenylpropanoids	0.00358
3	two-component signal transduction system (e.g. response regulator component)	2.18E-06
4	Energy	5.30E-14
5	Cell Wall	9.78E-11
6	Energy	6.90E-06
7	None	NA
8	biosynthesis of asparagine	0.04617
9	Energy	1.99E-11
10	Detoxification	0.00032
11	None	NA
12	Transport Mechanism	0.01944
13	Cell Wall	0.03601
14	Disease, virulence and defense	0.00177
15	Amino Acid Biosynthesis	0.00403
16	Storage Protein	0.01905
17	None	NA
18	Ribosomal Proteins	6.58E-13
19	biosynthesis of secondary products derived from L-phenylalanine and L-tyrosine	0.03256
20	None	NA

Additional Table 3. N-bicluster 9 gene list. Indicated here are the AGI identification for each gene, the TAIR 8 gene annotation for gene function and whether the gene is present in the initial 5 N/H biclusters considered in this analysis.

AGI	TAIR 8 Annotation	Significant N/H-bicluster Membership
**At1g78050**	phosphoglycerate/bisphosphoglycerate mutase family protein	NA
**At2g30670**	tropinone reductase, putative / tropine dehydrogenase, putative	NA
**At2g42350**	zinc finger (C3HC4-type RING finger) family protein	NA
**At5g48850**	male sterility MS5 family protein	NA
**At3g46130**	MYB111 (myb domain protein 111)	NA
**At2g48080**	oxidoreductase, 2OG-Fe(II) oxygenase family protein	NA
**At4g38340**	RWP-RK domain-containing protein	NA
**At1g16170**	similar to unknown protein	NA
**At1g48600**	phosphoethanolamine N-methyltransferase 2, putative (NMT2)	NA
**At5g67420**	LBD37 (LOB DOMAIN-CONTAINING PROTEIN 37)	NA
**At4g31910**	transferase family protein	NA
**At5g04950**	nicotianamine synthase, putative	NA
**At5g06570**	hydrolase	NA
**At5g19970**	similar to unknown protein	NA
**At5g62710**	leucine-rich repeat family protein / protein kinase family protein	NA
**At1g37130**	NIA2 (NITRATE REDUCTASE 2)	NA
**At5g66530**	aldose 1-epimerase family protein	NA
**At5g64550**	loricrin-related	NA
**At1g24280**	G6PD3 (GLUCOSE-6-PHOSPHATE DEHYDROGENASE 3)	NA
**At4g39770**	trehalose-6-phosphate phosphatase, putative	NA
**At1g60140**	ATTPS10 (TREHALOSE PHOSPHATE SYNTHASE)	NA
**At5g13110**	G6PD2 (GLUCOSE-6-PHOSPHATE DEHYDROGENASE 2)	NA
**At4g38470**	protein kinase family protein	NA
**At5g40850**	UPM1 (UROPHORPHYRIN METHYLASE 1); uroporphyrin-III C-methyltransferase	NA
**At3g17510**	CIPK1 (CBL-INTERACTING PROTEIN KINASE 1)	NA
**At5g50200**	WR3 (WOUND-RESPONSIVE 3); nitrate transmembrane transporter, NRT3.1	NA
**At3g16560**	protein phosphatase 2C-related / PP2C-related	NA
**At3g50900**	similar to unknown protein	NA
**At1g70410**	carbonic anhydrase, putative / carbonate dehydratase, putative	NA
**At1g43710**	EMB1075 (EMBRYO DEFECTIVE 1075); carboxy-lyase	NA
**At5g37260**	CIR1/RVE2 (CIRCADIAN 1)	NA
**At4g40070**	protein binding / zinc ion binding	NA
**At2g30040**	MAPKKK14 (Mitogen-activated protein kinase kinase kinase 14)	NA
**At1g08650**	PPCK1 (PHOSPHOENOLPYRUVATE CARBOXYLASE KINASE)	NA
**At4g09620**	similar to unknown protein	NA
**At2g16060**	AHB1 (ARABIDOPSIS HEMOGLOBIN 1)	NA
**At1g74030**	enolase, putative	NA
**At4g37610**	BT5 (BTB and TAZ domain protein 5)	NA
**At5g57660**	zinc finger (B-box type) family protein	NA
**At3g03040**	F-box family protein	NA
**At1g73920**	lipase family protein	NA
**At1g80440**	kelch repeat-containing F-box family protein	NA
**At1g25550**	myb family transcription factor	NA
**At4g24620**	PGI1 (CHLOROPLASTIC PHOSPHOGLUCOSE ISOMERASE)	NA

**At1g21010**	similar to unknown protein	NA
**At1g23870**	ATTPS9 (Arabidopsis thaliana trehalose-phosphatase/synthase 9)	NA
**At1g08090**	ATNRT2:1 (Arabidopsis thaliana high affinity nitrate transporter 2.1)	NA
**At3g52360**	similar to unknown protein	NA
**At5g07680**	ANAC079/ANAC080/ATNAC4 (Arabidopsis NAC domain containing protein 79, Arabidopsis NAC domain containing protein 80)	NA
**At4g34750**	auxin-responsive protein, putative / small auxin up RNA (SAUR_E)	NA
**At5g15830**	ATBZIP3 (ARABIDOPSIS THALIANA BASIC LEUCINE-ZIPPER 3)	NA
**At5g47560**	ATSDAT/ATTDT (TONOPLAST DICARBOXYLATE TRANSPORTER)	NA
**At1g62180**	APR2 (5'ADENYLYLPHOSPHOSULFATE REDUCTASE 2)	NA
**At1g22500**	zinc finger (C3HC4-type RING finger) family protein	NA
**At1g67920**	similar to unknown protein	NA
**At1g30510**	ATRFNR2 (ROOT FNR 2); oxidoreductase	NA
**At1g80310**	sulfate transmembrane transporter	NA
**At5g53460**	GLT1 (NADH-dependent glutamate synthase 1 gene)	NA
**At5g45340**	CYP707A3 (cytochrome P450, family 707, subfamily A, polypeptide 3); oxygen binding	NA
**At5g54970**	similar to unknown protein	NA
**At3g13560**	glycosyl hydrolase family 17 protein	NA
**At5g65010**	ASN2 (ASPARAGINE SYNTHETASE 2); asparagine synthase (glutamine-hydrolyzing)	NA
**At2g41310**	ATRR3 (RESPONSE REGULATOR 3); transcription regulator	NA
**At3g04530**	PPCK2 (PHOSPHOENOLPYRUVATE CARBOXYLASE KINASE 2)	NA
**At2g41560**	ACA4 (AUTO-INHIBITED CA(2+)-ATPASE, ISOFORM 4); calcium-transporting ATPase/ calmodulin binding	NA
**At3g14940**	ATPPC3 (PHOSPHOENOLPYRUVATE CARBOXYLASE 3)	NA
**At1g27980**	pyridoxal-dependent decarboxylase family protein	NA
**At4g26130**	similar to unknown protein	NA
**At5g41670**	6-phosphogluconate dehydrogenase family protein	NA
**At5g51460**	ATTPPA (Arabidopsis thaliana trehalose-6-phosphate phosphatase)	NA
**At4g34560**	similar to unknown protein	NA
**At1g04770**	male sterility MS5 family protein	NA
**At3g28510**	AAA-type ATPase family protein	NA
**At1g31770**	ABC transporter family protein	NA
**At3g13730**	CYP90D1 (CYTOCHROME P450, FAMILY 90, SUBFAMILY D, POLYPEPTIDE 1)	NA
**At3g19030**	similar to unknown protein	NA
**At4g39780**	AP2 domain-containing transcription factor, putative	NA
**At2g28890**	PLL4 (POLTERGEIST LIKE 4); protein serine/threonine phosphatase	NA
**At1g12110**	NRT1.1 (NITRATE TRANSPORTER 1.1)	NA
**At1g31200**	ATPP2-A9 (Phloem protein 2-A9); carbohydrate binding	NA
**At2g40750**	WRKY54 (WRKY DNA-binding protein 54)	NA
**At1g30270**	CIPK23 (CBL-INTERACTING PROTEIN KINASE 23)	NA
**At5g62900**	similar to unknown protein	NA
**At5g54660**	heat shock protein-related	NA
**At4g36380**	ROT3 (ROTUNDIFOLIA 3); oxygen binding / steroid hydroxylase	NA
**At5g13420**	transaldolase, putative	1
**At5g12860**	DIT1 (DICARBOXYLATE TRANSPORTER 1); oxoglutarate:malate antiporter	1
**At4g05390**	ATRFNR1 (ROOT FNR 1); oxidoreductase	1
**At2g27510**	ATFD3 (FERREDOXIN 3); electron carrier	1
**At2g36580**	pyruvate kinase, putative	1
**At4g26970**	aconitate hydratase, cytoplasmic, putative / citrate hydro-lyase/aconitase, putative	1
**At1g64190**	6-phosphogluconate dehydrogenase family protein	1
**At1g63940**	monodehydroascorbate reductase, putative	1
**At1g77760**	NIA1 (NITRATE REDUCTASE 1)	1
**At3g60750**	transketolase, putative	1

**At1g68670**	myb family transcription factor	6
**At3g24520**	AT-HSFC1 (Arabidopsis thaliana heat shock transcription factor C1)	6
**At2g17820**	ATHK1 (HISTIDINE KINASE 1)	6
**At3g49940**	LBD38 (LOB DOMAIN-CONTAINING PROTEIN 38)	16
**At1g73600**	phosphoethanolamine N-methyltransferase	16
**At4g34760**	auxin-responsive family protein	16
**At2g15620**	NIR1 (NITRITE REDUCTASE); ferredoxin-nitrate reductase	19
**At4g36010**	pathogenesis-related thaumatin family protein	19
**At1g64140**	similar to unknown protein	19
**At4g34740**	ATASE2 (GLN PHOSPHORIBOSYL PYROPHOSPHATE AMIDOTRANSFERASE 2); amidophosphoribosyltransferase	19
**At5g39590**	similar to unknown protein	19
**At5g42990**	UBC18 (UBIQUITIN-CONJUGATING ENZYME 18)	19
**At3g58610**	ketol-acid reductoisomerase	19
**At1g74660**	MIF1 (MINI ZINC FINGER 1)	20
**At4g18340**	glycosyl hydrolase family 17 protein	20
**At1g15550**	GA4 (GA REQUIRING 4); gibberellin 3-beta-dioxygenase	20
**At4g31730**	GDU1 (GLUTAMINE DUMPER 1)	20
**At1g78000**	SULTR1;2 (SULFATE TRANSPORTER 1;2)	20
**At5g01340**	mitochondrial substrate carrier family protein	1,6
**At5g35620**	LSP1 (LOSS OF SUSCEPTIBILITY TO POTYVIRUSES); RNA binding / translation initiation factor	1,16
**At3g02910**	Identical to UPF0131 protein	1,20
**At2g26980**	CIPK3 (CBL-INTERACTING PROTEIN KINASE 3)	6,19
**At1g70780**	similar to unknown protein	6,19
**At2g43760**	molybdopterin biosynthesis MoaE family protein	6,19
**At3g46640**	PCL1 (PHYTOCLOCK 1)	6,19
**At4g02920**	similar to unknown protein	16,19
**At1g32920**	similar to unknown protein	16,19
**At2g16660**	nodulin family protein	16,20
**At4g02380**	SAG21 (SENESCENCE-ASSOCIATED GENE 21)	16,20
**At5g03380**	heavy-metal-associated domain-containing protein	19,20
**At5g51830**	pfkB-type carbohydrate kinase family protein	19,20

Additional Table 4: Results of N/H Biclustering. Biclustering results for nitrate responsive genes from N-bicluster 9 biclustered over hormone treatment conditions. Rows highlighted in yellow met the criterion of having >50% reproducibility across bicluster experiments and a >1.5 fold change over reproducible bicluster experiments. The table indicates the bicluster number, the number of experiments; the percentage of those experiments that were replicates of each other (e.g. reproducible response); the number of genes in the bicluster; a description of the experimental treatment conditions supported by replicate experiments in the bicluster (with the genotype of the treated plants in parenthesis); and the tissue in which the treatment was performed. Details on the treatment conditions can be found on the NASC website (Craigon et al., 2004).

Bicluster	# of Expt	# Rep. Expt	# of Genes	Hormone Treatment Replicated in Bicluster	Tissue
20	6	83.33%	11	Cytokinin Treatment (WT and ARR10/12 DM)	Roots
6	7	71.43%	8	Cytokinin Treatment (WT and ARR10/12 DM)	Roots
16	9	55.56%	8	Cytokinin (WT and ARR10/12 DM)	Shoots
17	8	50.00%	11	Methyl Jasmonate (WT)	Seedlings
1	4	50.00%	13	ABA (WT)	Seed(Imbibition Stage)
19	4	50.00%	15	ABA (WT)	Seed(Imbibition Stage)
11	16	37.50%	5	Brassinolide(det2-1 mutant), Brassinosteroids (WT)	Seedlings
4	8	37.50%	8	Cytokinin Treatment (WT)	Seedlings
7	11	36.36%	8	Cytokinin Treatment (AtIPT8/pga22 Gain of Function Mutant)	Root Apex
21	11	36.36%	5	Cytokinin(ARR10/12 DM), Ethylene Inhibitors (WT)	Shoots, Seedlings
12	12	33.33%	7	Cytokinin Treatment (AtIPT8/pga22 Gain of Function Mutant)	Root Apex
5	6	33.33%	8	Cytokinin Treatment (ARR22-OX)	Seedlings
18	21	28.57%	5	Brassinolide (WT), Brassinosteroids (WT)	Seedlings
15	7	28.57%	8	IAA (WT)	Seedlings
9	33	24.24%	5	Brassinosteroids (WT), Ibuprofen (WT), GA3 (ga1-5 mutant)	Seedlings
3	20	20.00%	5	Cytokinin Treatment (AtIPT8/pga22 Gain of Function Mutant), GA3 Inhibitors (WT)	Root Apex, Seedlings
13	41	19.51%	5	Cytokinin(WT and ARR10/12 DM), Brassinosteroids (WT), Daminozide (WT), GA3 Inhibitors (WT)	Shoots, Seedlings
0	31	19.35%	6	Brassinosteroids (WT), Ethylene Inhibitors (WT)	Seedlings
14	31	19.35%	6	Daminozide (Ethylene Inhibitor), Salicylic Acid (Methyl Jasmonate Inhibitor), Auxin Inhibitor (WT)	Seedlings
10	11	18.18%	8	Cytokinin Treatment (WT)	Seedlings
2	13	15.38%	6	IAA (WT)	Seedlings
8	6	0.00%	10	NA	NA

Additional Table 5: Significant H-biclusters Over-represented Functional Categories. Comparison of functional representation higher frequency in the H-biclusters gene list when compared to the full N-bicluster 9 frequency as a background.

Bicluster	Significant Term(s)	P-value
1	ENERGY	0.010743
	Pentose phosphate pathway	0.038966
	C compound and carbohydrate metabolism	0.034179
	PLASTID	0.018203
	PHOTOSYNTHESIS	0.043716
	BIOGENESIS OF CELLULAR COMPONENTS	0.018203
	UNCLASSIFIED PROTEINS	0.036409
6	None	NA
16	CLASSIFICATION NOT YET CLEAR CUT	0.017727
19	PROTEIN FATE	0.03613
20	None	NA

Additional Table 6: Hormone Microarray Datasets: A list of all NASC (Craigon et al 2004) microarray datasets used for N/H biclustering. Indicated in this table is the description of the dataset from the NASC database, the number of chips in the total microarray dataset and the author of the microarray dataset. Only those experiments having to do with hormone treatments or relative control treatments were used in this study.

NASC Microarray Dataset Name	# of Chips	Reference
AtGenExpress: ACC time course in wildtype seedlings	12	Yoshida
AtGenExpress: Zeatin time course in wildtype seedlings	12	Yoshida
AtGenExpress: Methyl Jasmonate time course in wildtype seedlings	12	Yoshida
AtGenExpress: IAA time course in wildtype seedlings	12	Yoshida
AtGenExpress: ABA time course in wildtype seedlings	12	Yoshida
AtGenExpress: GA3 time course in wildtype and ga1-5 mutant seedlings	24	Yoshida
AtGenExpress: Brassinolide time course in wildtype and det2-1 mutant seedlings	24	Yoshida
AtGenExpress: Effect of Brassinosteroids on seedlings	26	Yoshida
AtGenExpress: Cytokinin treatment of seedlings	12	Mizuno
AtGenExpress: Effect of ABA during seed imbibition	8	Kamiya & Nambara
AtGenExpress: Basic hormone treatment of seeds	12	Yamaguchi
AtGenExpress: Effect of gibberellic acid inhibitors on seedlings	20	Yoshida
AtGenExpress: Effect of auxin inhibitors on seedlings	10	Yoshida
AtGenExpress: Effect of brassinosteroid inhibitors on seedlings	12	Yoshida
AtGenExpress: Effect of ethylene inhibitors on seedlings	6	Yoshida
AtGenExpress: Effect of ibuprofen, salicylic acid and daminozide on seedlings	8	Yoshida
Cytokinin treatment on Roots and Shoots	24	Sakakibara
Gibberellin and ethylene cross-talk	18	De_Grauwe
Auxin/cytokinin crosstalk in the Arabidopsis primary root apex	14	Ljung
