# Supplementary material for: A system biology approach highlights a hormonal enhancer effect on regulation of genes in a nitrate responsive "biomodule"
Source: BMC Syst Biol. 2009 Jun 6;3:59. doi: 10.1186/1752-0509-3-59 (PMC2702358; doi:10.1186/1752-0509-3-59)
Supplement: Additional file 2 — ANOVA [R] script and results. Contains the entire commented procedure of the ANOVA analysis, comparing N/H controlled genes vs. N-exclusive genes. [file 1752-0509-3-59-S2.pdf]

```
###########################################################################
#######
R output for ANOVA Model Simplification based on the Crawley Method
(Crawley 2005)
###########################################################################
#######




##Important Note:  All overlapping genes from the N/H biclusters in the
group factor were removed before this analysis was done


> NRF <- as.data.frame(read.table("NR_data_for_R.txt", header =
TRUE))## Data file with NR double mutant data (Wang et al 2004)
>
> NRF$Treament<-factor(NRF$Treatment)##Defning treatment factor
> NRF$Mutant<-factor(NRF$Mutant)## Defining Genotype Factor
> NRF$Group<-factor(NRF$Group)## Defining N/H-bicluster Factor
> NRF$Tissue<-factor(NRF$Tissue)## Defining Tissure Factor
> attach(NRF)## attaching the data frame to the R workspace
> levels(Treatment)
[1] "Treated"  "Control"
>levels(Mutant)
[1] "Plus"  "Minus"## Plus = mutant, Minus= WT
>levels(Group)
[1] "0"  "1"  "16" "19" "20" ## where 1, 16, 19 and 20 corresponde to
N/H ##biclusters 1, 16, 19, and 20 and 0 corresponds to N-bicluster 9
exclusive ##genes
>levels(Tissue)
[1] "Shoots"  "Roots"

##First model showing all the factors of interest for this analysis


> lmout<-lm(Expression~Group+Treatment+Tissue+Mutant+Group:Treatment)##
Defining the ANOVA model
> summary.lm(lmout)## performing ANOVA using the model defined above

##Results For First Model, note the high p-values for the tissue and
mutant ##categories.  The estimate is the coefficient estimate, the
error is the ##standard error, the t-value gives the t-statistic for
the null hypothesis of ##coeff = 0 and the Pr(>|t|) gives the p-value
based on the t-value.  Each ##factor level uses a baseline or control
(defined as the lowest number or ##letter for each comparison.
Interactions are over and above the main effects ##of the individual
factors.

Call:
lm(formula = Expression ~ Group + Treatment + Tissue + Mutant +
    Group:Treatment)

Residuals:
```

```
    Min      1Q  Median      3Q     Max
-5438.0 -1281.6  -421.1   153.9 23283.4

Coefficients:
                         Estimate Std. Error t value Pr(>|t|)
(Intercept)               603.31     237.53   2.540 0.011273 *
Group1                   1733.79     424.95   4.080 4.95e-05 ***
Group6                     87.01     519.03   0.168 0.866912
Group16                   760.43     519.03   1.465 0.143287
Group19                   681.63     402.04   1.695 0.090378 .
Group20                   910.45     454.34   2.004 0.045411 *
N-Treatment          1376.46     277.44   4.961 8.53e-07 ***
Tissue              -264.43     189.39  -1.396 0.163034
Mutant                 59.54     189.39   0.314 0.753308
Group1:N-Treatment   2103.86     600.97   3.501 0.000489 ***
Group6:N-Treatment   -604.19     734.02  -0.823 0.410681
Group16:N-Treatment   990.19     734.02   1.349 0.177720
Group19:N-Treatment  1089.91     568.57   1.917 0.055599 .
Group20:N-Treatment   654.50     642.53   1.019 0.308681
---
Signif. codes:  0 '***' 0.001 '**' 0.01 '*' 0.05 '.' 0.1 ' ' 1

Residual standard error: 2718 on 810 degrees of freedom
Multiple R-Squared: 0.2167,     Adjusted R-squared: 0.2041
F-statistic: 17.23 on 13 and 810 DF,  p-value: < 2.2e-16


##Second model:  We simplify one step at a time in order to see the
effects of ##removing individual factors.  The mutant factor is removed
because its p-value ##is highest.

> lmout1<-lm(Expression~Group+Treatment+Tissue+Group:Treatment)
> summary.lm(lmout1)

Call:
lm(formula = Expression ~ Group + Treatment + Tissue + Group:Treatment)

Residuals:
    Min      1Q  Median      3Q     Max
-5467.8 -1268.6  -431.0   162.7 23253.6

Coefficients:
                         Estimate Std. Error t value Pr(>|t|)
(Intercept)               633.08     217.72   2.908 0.003739 **
Group1                   1733.79     424.72   4.082 4.90e-05 ***
Group6                     87.01     518.75   0.168 0.866839
Group16                   760.43     518.75   1.466 0.143065
Group19                   681.63     401.82   1.696 0.090199 .
Group20                   910.45     454.08   2.005 0.045291 *
N-Treatment          1376.46     277.28   4.964 8.41e-07 ***
Tissue              -264.43     189.29  -1.397 0.162800
Group1:N-Treatment   2103.86     600.64   3.503 0.000486 ***
Group6:N-Treatment   -604.19     733.62  -0.824 0.410421
Group16:N-Treatment   990.19     733.62   1.350 0.177478
```

```
Group19:N-Treatment  1089.91     568.26   1.918 0.055463 .
Group20:N-Treatment   654.50     642.17   1.019 0.308412
---
Signif. codes:  0 '***' 0.001 '**' 0.01 '*' 0.05 '.' 0.1 ' ' 1

Residual standard error: 2717 on 811 degrees of freedom
Multiple R-Squared: 0.2166,     Adjusted R-squared: 0.205
F-statistic: 18.68 on 12 and 811 DF,  p-value: < 2.2e-16
```

##Comparison of first and second model, showing that the new model is not significantly worse in its explanatory power than the model with additional factors (based on p-value 0.7533) and therefore the model simplification is justified

```
> anova(lmout,lmout1)
Analysis of Variance Table

Model 1: Expression ~ Group + Treatment + Tissue + Mutant +
Group:Treatment
Model 2: Expression ~ Group + Treatment + Tissue + Group:Treatment
  Res.Df        RSS  Df  Sum of Sq      F Pr(>F)
1    810 5985212769
2    811 5985943108  -1    -730339 0.0988 0.7533
```

##Third model with tissue factor removed from model 2 above due to its high
##p-value

```
> lmout2<-lm(Expression~Group+Treatment+Group:Treatment)
> summary.lm(lmout2)

Call:
lm(formula = Expression ~ Group + Treatment + Group:Treatment)

Residuals:
    Min      1Q  Median      3Q     Max
-5335.6 -1291.4  -419.8   181.9 23385.8

Coefficients:
                         Estimate Std. Error t value Pr(>|t|)
(Intercept)                500.86     196.18   2.553 0.010860 *
Group1                    1733.79     424.96   4.080 4.95e-05 ***
Group6                      87.01     519.05   0.168 0.866916
Group16                    760.43     519.05   1.465 0.143298
Group19                    681.63     402.05   1.695 0.090387 .
Group20                    910.45     454.35   2.004 0.045416 *
N-Treatment          1376.46     277.44   4.961 8.54e-07 ***
Group1:N-Treatment   2103.86     600.99   3.501 0.000489 ***
Group6:N-Treatment   -604.19     734.05  -0.823 0.410695
Group16:N-Treatment   990.19     734.05   1.349 0.177731
Group19:N-Treatment  1089.91     568.59   1.917 0.055606 .
Group20:N-Treatment   654.50     642.55   1.019 0.308695
---
```

```
Signif. codes: 0 '***' 0.001 '**' 0.01 '*' 0.05 '.' 0.1 ' ' 1

Residual standard error: 2718 on 812 degrees of freedom
Multiple R-Squared: 0.2147,     Adjusted R-squared: 0.204
F-statistic: 20.18 on 11 and 812 DF, p-value: < 2.2e-16

##Based on ANOVA results the model simplification seems justified.
Model ##simplification is continued combining factor levels in the N/H
bicluster ##factor begins to see if the model can be further simplified
by removing factor ##levels with high p-values.

> levels(Group)## showing group levels
[1] "0"  "1"  "6"  "16" "19" "20"
> GroupA=Group## defining a new N/H bicluster group factor levels by
merging ##levels that are not significantly different from each other.
We begin by ##joining levels 0 and 6 of the group factor to form level
06.
> levels(GroupA)[3]="0.6"## setting N/H bicluster 6 to the baseline as
it is ##most similar to the N-bicluster 9 exclusive
> levels(GroupA)##new levels for the group
[1] "0.6"  "1"  "16" "19" "20"

## Fourth ANOVA model using revised N/H bicluster factor group factor
levels. Some N/H biclusters are still not shown to be significant
> lmout3<-lm(Expression~GroupA+Treatment+GroupA:Treatment)
> summary.lm(lmout3)

Call:
lm(formula = Expression ~ GroupA + Treatment + GroupA:Treatment)

Residuals:
    Min      1Q  Median      3Q     Max
-5335.6 -1282.0  -426.9   176.1 23459.7

Coefficients:
                          Estimate Std. Error t value Pr(>|t|)
(Intercept)                  513.3      181.5   2.828 0.004803 **
GroupA1                     1721.4      418.2   4.116 4.24e-05 ***
GroupA16                     748.0      513.4   1.457 0.145530
GroupA19                     669.2      394.9   1.695 0.090546 .
GroupA20                     898.0      448.0   2.005 0.045339 *
N-Treatment            1290.1      256.7   5.026 6.17e-07 ***
GroupA1:N-Treatment    2190.2      591.4   3.703 0.000227 ***
GroupA16:N-Treatment   1076.5      726.1   1.483 0.138563
GroupA19:N-Treatment   1176.2      558.5   2.106 0.035507 *
GroupA20:N-Treatment    740.8      633.5   1.169 0.242624
---
Signif. codes:  0 '***' 0.001 '**' 0.01 '*' 0.05 '.' 0.1 ' ' 1

Residual standard error: 2717 on 814 degrees of freedom
Multiple R-Squared: 0.2137,     Adjusted R-squared: 0.205
F-statistic: 24.58 on 9 and 814 DF, p-value: < 2.2e-16
```

```
##Comparison of ANOVA models, showing that the new model is not
significantly ##worse in its explanatory power than the model with
additional factors (based ##on p-value 0.6004) and therefore the model
simplification is justified

> anova(lmout2,lmout3)
Analysis of Variance Table

Model 1: Expression ~ Group + Treatment + Group:Treatment
Model 2: Expression ~ GroupA + Treatment + GroupA:Treatment
  Res.Df        RSS  Df  Sum of Sq      F Pr(>F)
1    812 6000347497
2    814 6007891685  -2   -7544188 0.5105 0.6004

## Further model simplification of the N/H biclusters with N/H
biclusters 16 ##and 19 combined because their estimated main effects
are close to each other ##compared to the standard error of their means

> levels(GroupA)
[1] "0.6"  "1"  "16" "19" "20"
> GroupB=GroupA
> levels(GroupB)
[1] "0.6"  "1"  "16" "19" "20"
> levels(GroupB)[3:4]="1619"
> levels(GroupB)
[1] "0.6"    "1"    "1619" "20"

##Fifth ANOVA model with second revision of N/H bicluster factor.  This
model ##shows a statistically significant difference between all
comparisons except ##for N/H bicluster 20 given nitrate treatment

> lmout4<-lm(Expression~GroupB+Treatment+GroupB:Treatment)
> summary.lm(lmout4)

Call:
lm(formula = Expression ~ GroupB + Treatment + GroupB:Treatment)

Residuals:
    Min      1Q  Median      3Q     Max
-5335.6 -1282.0  -422.0   176.1 23459.7

Coefficients:
                            Estimate Std. Error t value Pr(>|t|)
(Intercept)                    513.3      181.3   2.831 0.004752 **
GroupB1                       1721.4      417.7   4.121 4.15e-05 ***
GroupB1619                     696.6      336.0   2.073 0.038466 *
GroupB20                       898.0      447.4   2.007 0.045078 *
N-Treatment               1290.1      256.4   5.032 5.98e-07 ***
GroupB1:N-Treatment       2190.2      590.7   3.708 0.000223 ***
GroupB1619:N-Treatment   1141.5      475.2   2.402 0.016515 *
GroupB20:N-Treatment      740.8      632.8   1.171 0.242051
---
Signif. codes:  0 '***' 0.001 '**' 0.01 '*' 0.05 '.' 0.1 ' ' 1
```

```
Residual standard error: 2713 on 816 degrees of freedom
Multiple R-Squared: 0.2137,     Adjusted R-squared: 0.2069
F-statistic: 31.68 on 7 and 816 DF,  p-value: < 2.2e-16


##Comparison of ANOVA models for the two different vestions of N/H
bicluster ##factor showing that the new model is not significantly
worse in its ##explanatory power than the model with additional factors
(based on p-value ##0.9906) and therefore the model simplification is
justified
> anova(lmout3,lmout4)
Analysis of Variance Table

Model 1: Expression ~ GroupA + Treatment + GroupA:Treatment
Model 2: Expression ~ GroupB + Treatment + GroupB:Treatment
  Res.Df        RSS  Df  Sum of Sq      F Pr(>F)
1    814 6007891685
2    816 6008030390  -2    -138706 0.0094 0.9906

##Further model simplification combining group factor levels for N/H
bicluster 20 with N/H ##biclusters 16 and 19
> GroupC=GroupB ## redefining a new N/H bicluster factor level
> levels(GroupC)## showing levels of new N/H bicluster factor level
[1] "0.6"    "1"    "1619" "20"
> levels(GroupC)[3:4]="16.19.20"## combining N/H bicluster 20 with 16
and 19
> levels(GroupC)
[1] "0.6"        "1"        "16.19.20"

##Sixth ANOVA model with revised N/H bicluster factor level, this is
the ##final model as further simplification did not seem necessary
given the ##results
> lmout5<-lm(Expression~GroupC+Treatment+GroupC:Treatment)
> summary.lm(lmout5)



Call:
lm(formula = Expression ~ GroupC + Treatment + GroupC:Treatment)

Residuals:
    Min      1Q  Median      3Q     Max
-5335.6 -1245.3  -433.3   156.5 23459.7

Coefficients:
                                Estimate Std. Error t value Pr(>|t|)
(Intercept)                        513.3      181.1   2.834  0.00471 **
GroupC1                           1721.4      417.3   4.125 4.08e-05
***
GroupC16.19.20                     761.8      294.7   2.585  0.00990 **
N-Treatment                  1290.1      256.1   5.037 5.82e-07 ***
GroupC1:N-Treatment          2190.2      590.1   3.712  0.00022 ***
GroupC16.19.20:N-Treatment   1011.9      416.7   2.428  0.01539 *
```

```
---
Signif. codes:  0 '***' 0.001 '**' 0.01 '*' 0.05 '.' 0.1 ' ' 1

Residual standard error: 2711 on 818 degrees of freedom
Multiple R-Squared: 0.2134,     Adjusted R-squared: 0.2086
F-statistic: 44.37 on 5 and 818 DF,  p-value: < 2.2e-16

> anova(lmout4,lmout5)## comparison of the final model with the
previous model showing that the new model is not significantly worse in
its explanatory power than the model with additional factors (based on
p-value 0.8502) and therefore the model simplification is justified

Analysis of Variance Table

Model 1: Expression ~ GroupB + Treatment + GroupB:Treatment
Model 2: Expression ~ GroupC + Treatment + GroupC:Treatment
  Res.Df        RSS  Df  Sum of Sq      F Pr(>F)
1    816 6008030390
2    818 6010420271  -2   -2389881 0.1623 0.8502



## The final model here has the greatest explanatory power with all
factors and ##factor levels called significant.  Therefore this model
is the simplest model ##that can be derived from the initial model.
```
